# Social impact projections for Qatar youth residents from 2022: The case of the IAAF 2019

**DOI:** 10.3389/fspor.2022.922997

**Published:** 2022-07-28

**Authors:** Wadih Ishac, Kamilla Swart

**Affiliations:** ^1^Physical Education Department, Qatar University, Doha, Qatar; ^2^Division of Engineering Management and Decision Sciences, College of Science and Engineering, Qatar Foundation, Hamad Bin Khalifa University, Doha, Qatar; ^3^School of Tourism and Hospitality, University of Johannesburg, Johannesburg, South Africa

**Keywords:** psychic income, social impact, international sport events, social exchange theory, attendance and non-attendance, 2022 FIFA World Cup, youth residents' perception

## Abstract

While sport is playing an increasingly important role in society in the Middle East, there has been limited research on the perceived social impact of the hosting of major international events in this region. This study evaluates the main factors affecting youth residents' perceptions of hosting major international sport events, by measuring the psychic income in particular, generated within subgroups shaping their support toward hosting these events. Psychic income refers to the emotional and psychological benefit residents perceive they receive from hosting an international sport event. The study is of significance within the context of residents' perceptions studies given that the large majority of residents in Qatar are non-Qataris. Furthermore, the youth were the target population for this study given that they have been identified as the custodians of the next generation and as an essential force in molding national development; and extends the few residents' perception studies in Qatar which comprised the general population. Using the 2019 IAAF Athletics World championships as an example, a framework by Kim and Walker was adopted. Data were collected from 316 university students' from different nationalities residing in Qatar; a month after the event took place. After conducting confirmatory factor analysis, this study was subject to structural equation modeling. Overall, the results show that the perceived impact on Qatari youth nationals was higher compared to Arab youth, and non-Arab youth, respectively. Likewise, the perceived impact was higher for females compared to males. By evaluating the psychic income received by youth from different nationalities residing in Qatar, this study provides decision-makers and organizers with a better understanding of the outcome generated from hosting major international sport events, and how they can leverage these going forward. Of importance is that even if youth residents do not attend the event in person, these events may still generate positive psychic income which is particular relevant to the 2022 FIFA World Cup given the limitations related to purchasing tickets. With Qatar establishing itself as a regional sport hub by attracting a diverse range of international sport events, a cumulative approach to understanding psychic income is recommended.

## Introduction

Countries in the Arabian Peninsula are bidding to host international sport events with an interest that can be related to political and socio-developmental reasons (Foley et al., 2012; Dorsey, 2014; Ishac, 2020). Qatar presents an interesting case in that it is a “relatively new actor in the international sport arena” (Reiche, 2014, p. 2). Qatar, like its neighbors, has hosted different types of sport events. In 2020, before the pandemic COVID-19, Qatar was supposed to host around 65 local and international sport events (The Peninsula, 2020). The main objective of Qatar has been to invest in the sport industry as a vehicle for global recognition and to achieve its geopolitical goals (Amara, 2005; Reiche, 2014; Al-Thani, 2021). Dorsey (2014) argued that investing in the sport sector will help governments in the Middle East diversify their economy away from the oil and gas industries, and to appeal to the international community, find international support, and receive assistance when needed. Furthermore, these investments will help the country improve the tourism sector and foster community development as an alternative long-term source of revenue (Henderson, 2006). Qatar's strategy to establish itself as an international and regional sport hub is aligned to the country's National Vision 2030 (QNV 2030). Scharfenort (2012) contends that QNV 2030 provides a framework for implementing national strategies that aim to enhance its positioning in the region as well as to address human, social, economic, and environmental development. The Sports Sector Strategy (SSS) (2011–2016), one of the 14 sector strategies integrated in to the National Development Strategy (NDS), developed by the Qatar Olympic Committee (2011) highlights many of the potential roles sport, and major international sport events in particular, can play in diversifying the economy. Additionally, in aligning the SSS to the NDS and QNV 2030, the role of sport in human development (healthy and active lifestyles), social development (community cohesion and international solidarity), economic development (diversify economy through sport events and sport-related services) and environmental development (utilizing sport to create environmental awareness and utilizing environmentally-friendly sport facilities and goods) is underscored (Qatar Olympic Committee, 2011). In order to achieve these objectives, there has also been an increased emphasis on increasing women's participation in sport (Golkowska, 2017), as well as developing the youth through sport as custodians of the future (Qatar Olympic Committee, 2011; Gulf Times, 2021).

Furthermore, HE Permanent Representative of Qatar to the United Nations Sultan al-Mansouri emphasized that sport is an essential element to protect the youth and promote socio-economic development in Qatar and globally (Gulf Times, 2021). Reiche (2014) in his analysis of why Qatar is investing so heavily in the sport sector, highlights Qatar's efforts in promoting domestic elite sporting success, international sport investments abroad and the hosting of sport mega-events.

Concerning the latter; while emerging nations, in particular, use large-scale sporting events as both foreign and domestic policy tools (Reiche, 2014) and anticipate impacts that surpass the event itself, there is much debate as to whether these events deliver long-term benefits for the host country. Moreover, although there is an increasing body of literature on the impacts and legacies of sport mega-events in the emerging context (Cornelissen et al., 2011; Swart and Bob, 2012; Brittain et al., 2018), there is limited research on these aspects in the Arab World (Al-Emadi et al., 2022). They further argue that insufficient research has been conducted on understanding residents' perceptions of the socio-cultural impacts of major international sport events in this region.

Oshimi et al. (2021) highlight that the social impact literature related to large scale sport events has increased since the 1980's, especially in relation to changes in the perceptions of the impacts pre- and post the event (Kim and Petrick, 2005; Balduck et al., 2011; Gibson et al., 2014). The influence of social impact on residents' intentions to support an event (Kaplanidou et al., 2013; Prayag et al., 2013; Parra-Camacho et al., 2020b); intention to host the event in the future (Liu, 2016; Oshimi et al., 2016; Parra-Camacho et al., 2020a); and to support sport policy (Parra-Camacho et al., 2020c), has also been investigated. More recently, Oshimi et al. (2021) contributed further to the literature by examining the relationships between perceived social impact (3 months before) and actual residents' viewing behavior (4 months after) by utilizing qualitative panel data to assess intention to support events based on the social impacts experienced at the 2019 Rugby World Cup in Japan.

Many researchers also claim that among the social benefits associated with hosting major sport events are psychological benefits to the host community. Crompton (2002) and Liu and Gratton (2010) argued that the social benefits of hosting international/mega sport events may be greater in the long run than the short-term economic impact generated by the influx of visitors to the host country during the event. Psychic income is one of the constructs that have been used to define psychological benefits. Several researchers assessed the “psychic income” generated from hosting sports events (Burgan and Mules, 1992; Gibson, 1998; Crompton, 2004; Owen, 2006; Kim and Walker, 2012; Gibson et al., 2014). Crompton (2004) investigated the host community's perception of the impact of participating/ attending a sport event, focusing particularly on identifying the intangible motivation behind such participation. To the authors' knowledge, few studies have assessed the social impact associated with hosting sport events in Qatar (Al-Emadi et al., 2017, 2022; Ishac et al., 2018). In their study, Ishac et al. (2018) assessed the psychic income generated after the event took place, while these other studies, were pre-event assessments. Moreover, while Al-Emadi, Sellami and Fadlalla (2022) study focused on the social and cultural impacts of 2022 FIFA World Cup on Qatar's community, also taking nationality into consideration; the present study extends this work on social impacts by focusing on the concept of psychic income specifically. Furthermore, the authors examined the psychic income associated with the hosting of 2019 IAAF (International Association of Athletics Federations) World Athletics Championships hosted by Qatar. This Championships is of significance as it was one of the last large scale international sport events to be hosted in Qatar prior to Covid-19.

A study by Gibson et al. (2014) on the psychic income and social capital derived from the hosting of the 2010 FIFA World Cup utilized the items developed and tested in the Australian event context by Fredline and Faulkner (2000). The four items, which they claim are consistent with the literature describing psychic income from mega-events, comprised that the event would “(1) increase community spirit and pride, (2) increase feelings of national pride and patriotism, (3) make people feel good about themselves and their community, and (4) help bring people together in celebration.” (Gibson et al., 2014, p. 117). Moreover, they contend that at the time of conducting their study a comprehensive measure of the construct was non-existent. Thus, Gibson et al. (2014) further suggest that future research could consider adapting Kim and Walker (2012) scale for use with sport mega-events.

In the current study, the measurement scale developed by Kim and Walker (2012) and modified to the Arabic language by Ishac et al. (2018) was adopted for use in this study. Hence, the questionnaire comprising five different dimensions (a) community pride/image, (b) community attachment, (c) event excitement, (d) community infrastructure, and (e) community excitement (Kim and Walker, 2012) was utilized. The authors also followed the modified definition of psychic income by Weight et al. (2019) that permitted residents who did not attend the event physically to also participate in the study.

In relation to its objectives, Qatar has focused on youth development through attracting them to become more engaged in sport activities which can motivate them to lead a healthier and productive lifestyle (Qatar Olympic Committee, 2011). Moreover, the General Secretariat for Development Planning (2012) specifically focuses on the youth in support of achieving QNV 2030. Once again, the role of sport as a tool for developing a health and cohesive society in underscored, along with the aim on increasing youth participation in sport. The focus on the youth is also of particular significance to Qatar in that 82.44% of the population is aged between 15 and 54 years (Index mundi, 2022). Furthermore, the nationality of residents within the context of previous residents' perceptions is rather unique in that Qatari nationals/citizens represent only 11.65% of the population, with the majority of residents in Qatar being non-Qatari citizens (World Population Review, 2022). Following Ma et al. (2013) and Wicker and Sotiriadou (2013) they identified that specific demographic factors (such as gender, age, and nationality) are more likely to express higher levels of psychic income. This study focuses on the nationalities and not the ethnicity of the participants for the reason that ethnicity is more related to the heritage and ancestry. The focus on nationality and not citizenship is because the state of Qatar has never been an immigration destination or long-term settlement (Babar, 2014). In fact, the presence of the majority of foreign workers in Qatar is a genuine and composite reflection of the socioeconomic environment demand. While Qatar has been highly reliant on foreign labor especially in relation to infrastructural development, the country has also been attracting highly skilled foreign workers in line with developing its knowledge economy. Qatar citizenship law has created a level of exclusion by injecting stringent criteria of eligibility to ensure citizenship remains off-limits to foreigners (Babar, 2014). Although Qatari nationality does not apply to any new baby born in the country as specific conditions need to be met (Hukoomi, 2022a), Babar (2014) emphasizes there are many from other Arab countries in particular that have resided in the country for decades. Thus, it is also important to highlight that 22 countries make up the Arab League which is a “voluntary association of countries whose peoples are mainly Arabic speaking or where Arabic is an official language” (BBC, 2017). It is evident that Qatar presents a unique socio-economic and cultural context in comparison to previous World Cups. Thus, this study provides further insights by categorizing Qatar youth residents into two major groups based on their nationalities as Arabs including all Arab countries except of Qatar nationality/ citizenship and non-Arabs. Thus within this categorization this study goes further and distinguishes between Qatar nationality/citizens and Arabs, for the reasons discussed above. As a result a third group were created; the first group is Qatar youth residents with Qatari nationality, the second one Qatar youth residents with Arab nationality excluding the Qatari national, and the third one is non-Arab youth nationals excluding Qatari and Arab nationals. This regrouping distinction was previously implemented by Ishac et al. (2018) and is used in this study to provide the comparative lens to examine psychic income within the context of the first World Cup in the Arab region.

Data were collected from the Qatar local community a month after the 2019 IAAF Championships took place. The target audience for this study was, specifically, the youth of Qatar as opposed to the previous residents' perception studies (Al-Emadi et al., 2017, 2022). This study further builds on Al-Emadi, Sellami and Fadlalla (2022) study, which underscores the importance of nationality within the Qatari context; however, they only considered two subgroups, viz. Qatari and expatriate residents. They further considered their study as pioneering, and according to their knowledge, the first study “that explores the importance of nationality on the perceived impact of mega events” (p. 3).

The results of this study show the impact of hosting international/mega sport events on different groups in society, which both academics and local sport event organizers can utilize. Based on the study findings, the host city, decision-makers, and authorities can evaluate the strategies that they employ when hosting international/mega sport events so these would have a greater impact on the host community, and consequently the objectives of the events could be attained. Such analysis can contribute to further understanding the impact associated with the hosting of the 2022 FIFA World Cup, which can be beneficial to both the host country in terms of future events as well as other countries within the Gulf since it can provide more insights into youth residents' behavior toward the events.

## Literature review

### Social exchange theory

Various benefits can be claimed to be generated from hosting major sport event before, during, or after the events; these benefits vary between physical, cultural, social, political, economic, and psychological (Gramling and Freudenburg, 2010). The majority of studies conducted highlighted that the social impacts of sport events are multi-dimensional including both positive and negative impacts (Ohmann et al., 2006; Balduck et al., 2011; Ma et al., 2013; Polcsik and Perényi, 2021), and vary based on the size of the event (Müller, 2015). Social exchange theory (SET) is one of the main theories that focus on assessing the perceived social impact mainly being residents' perceptions based on awareness, attitude, and intention (see Ap, 1992; Jurowski et al., 1997; Karadakis and Kaplanidou, 2012).

SET was described by Ap (1992, p. 668), as “a general sociological theory concerned with understanding the exchange of resources between individuals and groups in an interaction situation.” According to this theory, Gursoy and Kendall (2006) explain that the involvement of an individual or a group of people with another party will be based on the estimation that some benefits will result from such exchange. On this basis, active members of society apply the same approach to the perceived costs and benefits acquired from their participation in the event, and based on that they might or might not decide to participate. In this study, the participation benefits will be modified since this work assesses the perception of university students who have attended and/or are aware of this event.

SET is built on a psychological principle (Homans, 1961), and is measured by psychic income perceived by residents of the host country. Measuring the psychic income can provide decision-makers, with better awareness directing their investments toward hosting sport event thus generating a positive social and psychological impact within the society.

### Psychosocial benefits

Studies focusing on assessing the impact generated from hosting sport events have been focusing on the intangible benefits (Waitt, 2003; Kim and Petrick, 2005; Balduck et al., 2011; Kim and Walker, 2012; Slabbert and Oberholzer, 2012; Kim et al., 2014). Many research studies have argued in favor of separating the social impact from the psychological impact (Ritchie and Aitken, 1985; Burgan and Mules, 1992; Crompton, 2004); however, noting their correlation, it is difficult to separate them (Kim and Petrick, 2005; Kim et al., 2006). Subsequently, two ways exist to assess the social impact perceived by the residents (Faulkner and Tideswell, 1997). The first investigates on the macro level by evaluating the cultural and environmental aspects (Kim and Petrick, 2005). The second focuses on the psychological and emotional states of the residents (Waitt, 2003). Within the second subdivision, researchers have identified several constructs, such as feel-good effect (Maennig, 2008), wellbeing (Kavetsos and Szymanski, 2010), or happiness (Taks et al., 2015).

In their study, Mair et al. (2021) simplified the social impact to eight different key impact categories, the second being social cohesion, civic pride, and social capital. This category focuses on how people feel within their community (Duffy and Mair, 2017), and can be translated by the level of perceptions of wellbeing and quality of life within the society (Kaplanidou et al., 2016). Next to that civic pride is connected to the shared beliefs about the place, in other words the attitudes and the feelings of residents toward their local area (Wood, 2006), while social capital can be reflected by the expectation that can be seen or has arisen within the community (Gibson et al., 2014). This study falls under the second impact category as it focuses on assessing the perceived impact within the community, and project it into the future.

### Psychic income

Psychic income is one of the constructs that have been used to define the socio-psychosocial benefits generated by an individual (Burgan and Mules, 1992; Crompton, 2004; Wicker et al., 2012), as highlighted previously. For instance, in the sport context, Ritchie (1984) expressed that within the local community a sense of feeling of pride and enthusiasm can be associated to the international recognition associated with hosting international sport mega- events. This type of sentiments have been described as the “feel good factor” or psychic income (Burgan and Mules, 1992; Maennig, 2008; Cornelissen and Maennig, 2010).

Crompton (2004, p. 181) being one of the main researchers that used psychic income to measure the impact generated from a sport event, defined psychic income as “the emotional and psychological benefit residents perceive they receive, even though they do not physically attend sports events and are not involved in organizing them.” Crompton targeted the host community and their perceived impact from participation in the sporting event.

Building on his definition Crompton (2004) provided a specific framework to assess the psychic income within the community. His framework consists of seven components (a) community pride resulting from increased visibility, (b) civic pride from being a sports event host city, (c) pride in efforts to resuscitate deteriorated areas, (d) enhanced collective self-esteem, (e) tangible focus for social bonding, (f) excitement from the event visitors, and (g) emotional involvement with a sport event.

Based on this framework many researchers expounded on the nature of psychic income perceived from hosting sport events. For instance, 3 months after the 2002 World Cup, Kim et al. (2006) found a minor increase in community pride, while Gursoy et al. (2011) noted a minor decrease in Beijing residents 3 months after hosting the Olympic Games. On the contrary, Ndlovu-Gatsheni (2011) highlighted that the feeling of pride and patriotism improved within South Africa after hosting the FIFA World Cup. Furthermore, Kim and Walker (2012) used a five-factor model derived from Crompton's framework to assess residents' perception of Tampa Bay, Florida during Super Bowl XLIII. Their study focused on the perceived impact after the event took place, the five assessed components are: (a) community pride/image, (b) community attachment, (c) event excitement, (d) community infrastructure, and (e) community excitement. They recommended further development for the used scale by applying it on bigger sport events. Gibson et al. (2014) reiterated this call after conducting their study on the psychic income derived from the 2010 FIFA World Cup in South Africa. They added that at the time of conducting their study a comprehensive measure of the construct was non-existent, as mentioned previously.

Kim et al. (2015) assessed the psychic impact generated on the residents of the Korean Grand Prix. This study was built on a three-factor model determining positive and negative psychological impact (perceived economic benefits, perceived community pride, and perceived community development). Liu (2016) assessed the psychic impact perceived by the 2008 Olympic Games in Beijing. This study assessed six different models, (image and status, international exchange and cooperation, economic and tourism development, infrastructure development, inconvenience of life, and environment pollution and security concern). In addition, Ishac et al. (2018) assessed the psychic income associated with the 2015 Handball World Championship in Qatar, where they modified the type of the sport event to an international one, as recommended by Kim and Walker (2012).

More recently, Weight et al. (2019) modified the original definition of psychic income by Crompton (2004), where they replaced “do” by “may” to make the definition more inclusive targeting both fans and non-fans. As a result, the definition became: “the emotional and psychological benefits residents perceive they receive, even though they [may] not physically attend sports events, and are not involved in organizing them” (Weight et al., 2019, p. 134). Therefore, the main focus of this modified definition is to identify the intangible motivation behind residents' being part of this sport events, irrespective whether they attended or not.

## Conceptual framework

In the MENA region, to the author's knowledge, not many studies have assessed the psychic impact generated from hosting sport events (Ishac et al., 2018; Ghaderi et al., 2021). In Qatar, Ishac et al. (2018) assessed the perceived psychic income generated from hosting the Handball World Championships 2015 on residents in Qatar. In their study, they assessed and validated the five components developed by Kim and Walker (2012). Therefore, assessing the impact generated on Qatar's residents while focusing on subgroups within the society will provide valuable insight into decision-making. This leads to the following question: Did hosting the 2019 IAAF World Championship generate satisfaction amongst students' residing in Qatar regarding the social and psychic characteristics? And what projections could be made in relation to the hosting of the 2022 FIFA World Cup?

In 2019 Qatar hosted the IAAF World Athletics Championships, where they faced many challenges, among which the number of athletes withdrawing due to the heat, and the empty seats inside the stadium. For instance Knudsen et al. (2020) highlighted that for the Men's 100 m final, only 11,000 spectator showed up in Khalifa Stadium, and around 1,000 spectator for the Women's 100 m. Against this background, this study examines how the IAAF 2019 was perceived by the youth residents in Qatar. It explores whether this event played a role in accelerating the country's readiness for the 2022 FIFA World Cup, by assessing community support, amongst the youth, which is considered a key ingredient to the success of an event (Prayag et al., 2013; Kim et al., 2015). As demonstrated in this paper, psychic income can be gained by the host community even if they do not participate directly in the event through attendance.

One of the aims of QNV 2030 is to enhance developing the society through hosting and investing in the sport industry; therefore, the authors focused on measuring the impact by sub-dividing the population. Assessing and understanding the psychic income component within a smaller scale community (namely, the youth) is valuable for the cumulative understanding given the decision-maker/organizers approach of investing heavily in hosting international sport events, with the belief that they are beneficial to society as a whole. Moreover, local residents' support is integral to the success of the sporting events, as mentioned previously.

Many researchers believe that identifying the specific demographic factors (such as gender, age, and nationality) are more likely to express higher levels of psychic income (Ma et al., 2013; Wicker and Sotiriadou, 2013). Reviewing specific variables can help administrators identify which groups are more likely to be satisfied with the effect of hosting international sport events, as well as other groups that may need more active involvement to achieve higher satisfaction levels. In a similar context, Sheldon and Var (1984) and Brunt and Courtney (1999) found that the longer-term residents were more influenced compared to short-term residents. When it comes to residents from different nationalities or different groups within the society, studies found that the place where residents grew up, or the place of birth are important factors that influence residents' perceptions (Lankford, 1994; McCool and Martin, 1994; McGehee and Andereck, 2004). In their study, Wicker and Sotiriadou (2013, p. 29) found that locals are more likely to gain a positive attitude while assessing the impact generated from the 2006 Melbourne Commonwealth Games: “People of Aboriginal or Torres Strait Islander origin are more likely to spend more time participating in sport as a result of Melbourne hosting the Commonwealth games”.

With 88.4% of the residents of the country being non-Qatari (World Population Review, 2022), this study focuses on subgroups, categorizing participants by their nationalities, for instance, Qatari nationals as a group, Arabs excluding the Qataris as another group, and the third group as non-Arabs.

Next to nationality, gender is another factor that researchers assessed while measuring the perceived impact associated with hosting sports events. Findings show that hosting sport events will generate a more positive impact on females than males. For instance, assessing the impact of the Barcelona Olympic Games shows that females experienced a more significant impact compared to males (Truno, 1995). Furthermore, Kim and Petrick (2005) found that female respondents perceived the FIFA 2002 World Cup more positively. Similarly, concerning the 2006 Melbourne Commonwealth Games; Balduck et al. (2011) found that the opinion of female was higher compared to the male one; Wicker and Sotiriadou (2013) noted that females are more likely to gain a positive attitude. Moreover, Gibson et al. (2014), while assessing the psychic income associated with the FIFA World Cup in South Africa found that after the event took place, women showed a high level of pride and excitement which can be related to the event itself (the World Cup) since it achieves a “banalisation of nationalism” Brunt and Courtney (1999, p. 282). More recently, Storm and Jakobsen (2020) also observed that women are more proud of their country than men. On the other hand, a study published by Oshimi et al. (2016) show that gender did not effect the results. Hence, this study will focus on gender as a dependent variable, measuring if hosting sport events can promote the recent government initiative in promoting women's participation in sport (Golkowska, 2017).

**Figure 1:**
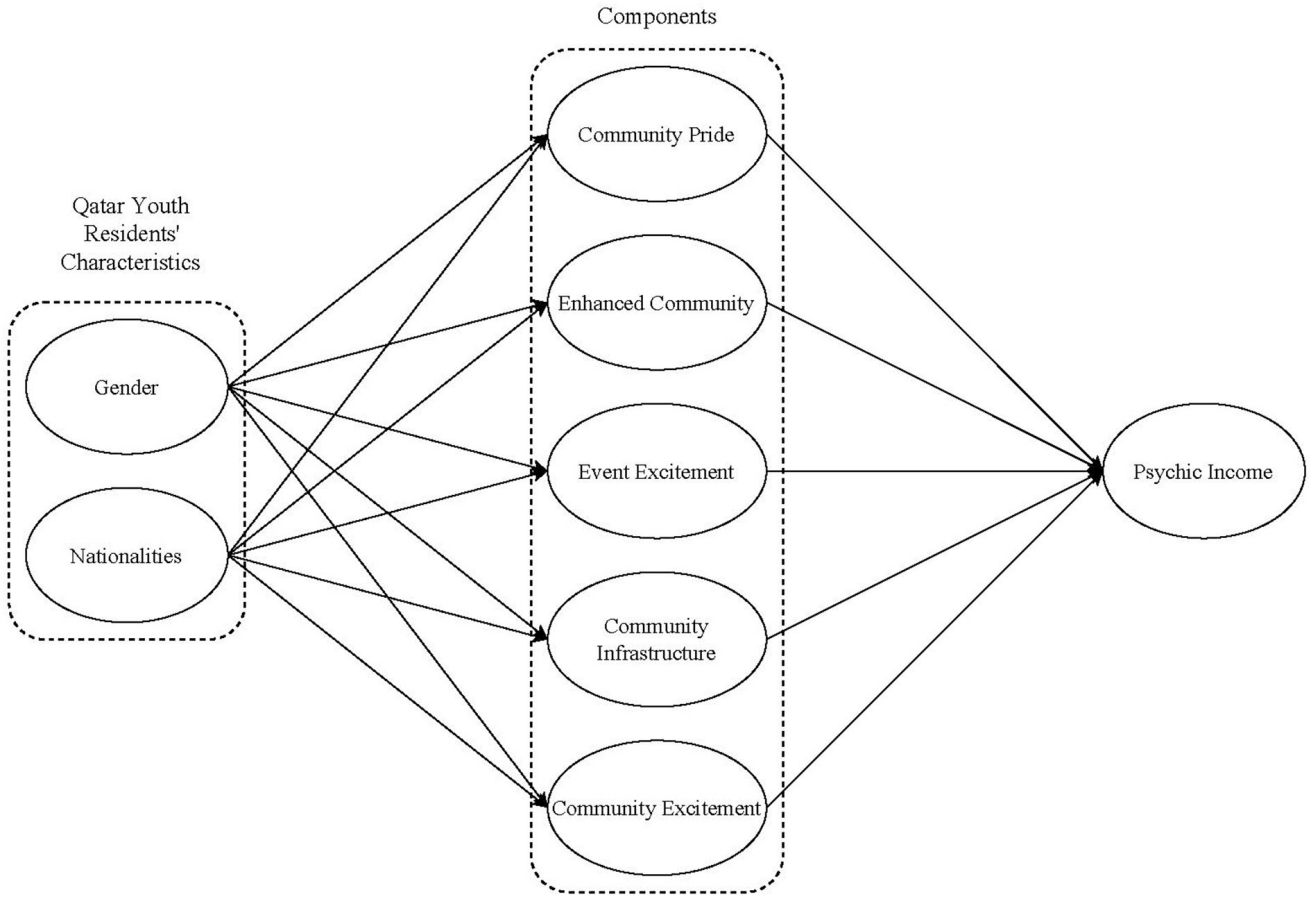
Conceptual model.

Thus, to summarize, it is clear that previous studies highlight that focusing on several demographic factors can provide a better understanding of residents' perceptions generated from hosting sport events. Based on the literature, the conceptual framework described in Figure 1 was used to examine the relationships between the different variables mainly by measuring the impact generated between the main five dimensions: Community pride, Enhanced community attachment, Pride to improve infrastructure, Event excitement, and Community excitement, in respect to the psychic income. To overcome this research gap, the following overall hypotheses were formulated and tested:

*Hypothesis 1*: Hosting the 2019 IAAF World championships will have a greater psychic impact on Qatari youth nationals compared to non-Qatari youth nationals.
*Hypothesis 2*: Hosting the 2019 IAAF World championships will have a greater psychic impact on female youth in Qatar compared to male youth.

While previous research has indicated that the following demographic variables: nationality, gender and age are more likely to express higher levels of psychic income; this study did not include age given that the focus of the study is on the psychic income of youth in Qatar in relation to the hosting of the 2019 IAAF World Championship.

## Methodology

### Item generation

The study followed the framework adopted by Kim and Walker (2012) which was modified to the specific sport event assessed, and translated to the Arabic language by Ishac et al. (2018), as mentioned previously, to measure the psychic impact of the 2019 IAAF Athletics World Championships on university students.

The measurement focused on the 5 dimensions mentioned in Figure 1, with a total of 30 questions distributed as followed: 7 items addressing community pride, 9 items addressing community attachment, 6 items addressing event excitement, 5 items addressing community infrastructure, and 3 items addressing community excitement (Crompton, 2004; Kim and Walker, 2012; Kim et al., 2015; Liu, 2016). The 30 questions were assessed through a seven-point Likert scale, that ranged from 1 (strongly disagree) to 7 (strongly agree); the scale assisted in measuring the participants' attitudes toward each of the questions.

### Sampling

This work attempts to measure the impact of hosting the IAAF 2019 Athletics Championships on the host city's youth community. Accordingly, the sample focuses on university students being the generation that will reshape the future of the country, as highlighted previously. As a result, Qatar University (QU) the largest university in Qatar was chosen. QU has around 23,000 students across nearly 85 different nationalities (Qatar University, 2021). Furthermore, QU has the highest number of Qatari university-level students. In a Fact Book published by QU, it is highlighted that from the student body, 14,854 are Qataris (64%), while 7,607 are non-Qataris while 5,040 being male and 17,421 (75.7%) are female (FACT Book, 2020). Any result that falls under a similar percentage will be representative (Omair, 2014). Furthermore, this information will help the present study to better assess the impact of the IAAF event on students from different nationalities.

### Data collection

The questionnaire written by one of the co-authors, who speaks English and Arabic fluently, was sent to be assessed by experts in the field for their input. Subsequently, a pilot test was conducted by university students, and the items were modified following their feedback. Based on the results, the final version included the questions in both English and Arabic languages and was sent to the Institutional Review Board for ethical approval. Following the approval, and 1 month after the 2019 IAAF World championships took place, the questionnaire was uploaded online. An email was sent to QU students explaining the purpose of the study, directing them to a link to the survey. One month later, and after having removed the incomplete surveys, 316 were returned. Of the respondents, 62% were Qataris (*n* = 194), while almost 75% were female Qataris (*n* = 236); thus making our sample representative.

### Data analysis

The study follows a confirmatory factor analysis (CFA) approach to measure the psychic income associated with the 2019 IAAF Athletics World championships. First, the survey was tested for validity and reliability, it was important to validate the association between survey items and their corresponding factors (Kim and Walker, 2012; Liu, 2016). Next, Cronbach's Alpha and Composite Reliability (CR) were applied, among others, to measure the internal consistency reliability. Second, the study used Structural Equation Modeling (SEM), to describe the association between the dimensions. Furthermore, this study attempted to find the association between the five dimensions (the psychic income) and the gender and nationality and the association between the five dimensions and the overall psychic income (see Figure 1). Therefore, when testing theories that include latent variables (multiple items under each dimension) SEM will do all the different tests together at the same time, this will lead to a reduction in the Type One error while the statistical power will increase. As a result, the Variance Inflation Factor values were examined to perform the collinearity analysis. The goodness of fit of the structural model has also been assessed among several fit indices. After that, the Q-square was assessed to measure the model's predictive accuracy (Hair et al., 2016). Finally, to test the hypotheses, a structural path was used to measure the path coefficients, standard error, t-values, and *p-*values.

It is also worth noting that in the repeated indicators approach, the manifest indicators of the first-order constructs (the five dimensions) are reused for the second-order construct (the psychic income); this procedure was suggested by Wold (1982). In essence, in this approach a second-order construct is directly measured by using all of the first-order common factors' manifest variables. In this case, the psychic income in our study is not a single scale variable; instead, it is measured using 30 questions. The margin of sampling error of this study is ±5.48% following Krejcie and Morgan (1970).

## Results

### Measurement model: CFA

Data generated from hosting the 2019 IAAF World Championship were submitted to a CFA analysis, where the internal reliability of each of the items was measured. According to Kline (2005), a standardized factor loading should be above 0.7. All of the factor loadings were greater than the suggested standard except one item (EE7: 0.679) was removed from the final version.

For internal consistency (Netemeyer et al., 2003) Cronbach's Alpha and CR were measured. A value of 0.7 or above is considered acceptable for both Cronbach's Alpha and composite reliability (Hair et al., 2016). Table 1 shows that the range of Cronbach's Alpha is between 0.844 and 0.934, while the range of composite reliability values is between 0.908 and 0.941 implying that the results are acceptable.

**Table 1:** Validity and reliability of the questionnaire.

**Variables**	**Mean**	**SD**	**Cronbach's Alpha**	**AVE**	**CR**
***CP- Community pride***			0.844	0.621	0.920
CP1 Gained a positive image as host city	6.354	1.219			
CP2 Gained positive recognition	6.342	1.097			
CP3 Showed ability to host major sport event	6.196	1.250			
CP4 IAAF gave opportunity to showcase the city	6.386	1.172			
CP5 Event helped my community to become a nationally known city	6.263	1.170			
CP6 Qatar can host other major sport events	4.867	2.005			
CP7 Hosting the event allowed for outsiders know more about my community	5.206	1.776			
***EC- Enhanced community***			0.943	0.666	0.941
EC1 Strengthened my friendships in my community	5.396	1.763			
EC2 Made residents appreciate their way of life more	5.127	1.875			
EC3 Increased my sense of wellbeing	5.044	1.830			
EC4 Increased my sense of belonging in various community groups	5.620	1.554			
EC5 Increased social interactions within community	5.266	1.745			
EC6 Watching the IAAF increased my respect to the community	5.658	1.534			
EC7 Increased cooperation among groups in my community	5.345	1.783			
EC8 Increased my community confidence	5.013	1.947			
***EE- Event excitement***			0.907	0.718	0.939
EE1 Hosting the IAAF increased my interest in the athletics	4.858	2.080			
EE2 Watching the tournament increased my fan involvement with Athletics	5.288	1.802			
EE3 I enjoyed watching more the Athletics games	5.117	1.879			
EE4 During the tournament the night life was more exciting	5.519	1.736			
EE5 When attending the tournament I enjoyed interacting with visitors	5.756	1.497			
EE6 I liked to watch the IAAF championships	5.753	1.497			
***PE- Pride community infrastructure***			0.923	0.664	0.908
PE1 Hosting the tournament improved the quality of community public services	6.032	1.224			
PE2 Holding the 2019 IAAF improved the quality of police and fire services	5.715	1.412			
PE3 Accommodating the event improved our public facilities	5.769	1.430			
PE4 Organizing international sport event promoted opportunities to revive the community	5.854	1.337			
PE5 Presenting the intenrnational sport competition helped urban regeneration	5.886	1.340			
***CE- Community excitement***			0.893	0.788	0.917
CE1 Hosting the event provided entertainment to the community	5.867	1.312			
CE2 The tournament brought excitement to the community	4.358	0.910			
CE3 Holding the championship provided new activities to the community	4.215	0.883			

For a better understanding of the applied scales correlation to the other variables, the Average Variance extracted (AVE) test was applied. Based on Fornell and Larcker (1981), for a validated scale, the AVE value should be greater or equal to 0.5. As a result, the assessed scale shows values between 0.621 and 0.788, meaning that the used scale questionnaire in this study is validated consistency missing so, change “(see Table 1).”

### SEM and model fit indices

In order to apply SEM it is important to make sure of the validity of the sample. Following Kline, (2015) who cautions that when the sample is not large this may amplify the standard error, thus he highlights that a median sample size maybe around 200 cases (p. 16). In our case, the sample size is 316 making our sample valid to use SEM. Following this validation, the SEM was applied using STATA 16 as software for statistical analysis. Result shows a satisfaction between the model and the observed data. According to Cangur and Ercan (2015), an SRMR value < 0.08 is considered a good fit. Following, Smith and McMillan (2001), if the CFI value is >0.9, means the model is fit, furthermore, based on Marsh and Hocevar (1985), χ2/df values between 2 and 5 denote a reasonable model fit. Our model shows (χ2/df 3.371; SRMR for the final model is 0.068, and the comparative fit index (CFI) of the final model was 0.900).

The next step showed that the structural path model has minimum collinearity since the values of the Variance Inflation Factor are below five (Sarstedt et al., 2017). Overall, the observation of the predictive relevance (Q∧2) was equal to 0.528, which indicated that the structural model was acceptable being greater than the cut-off value of zero (Hair et al., 2016). Table 2 shows the path coefficients, standard errors, T-values, and *P*-values.

**Table 2:** Structural model results.

**Structural paths**	**B**	**SE**	**t**	**p**
**Total residents**				
Community excitement -> Psychic income	0.137	0.005	27.965	0.000
Community pride -> Psychic income	0.221	0.018	12.090	0.000
Enhanced community attachment -> Psychic income	0.338	0.014	24.353	0.000
Event excitement -> Psychic income	0.259	0.012	20.757	0.000
Pride to improve infrastructure -> Psychic income	0.210	0.007	30.448	0.000
**Subgroup by nationality**				
Nationalities (Qatari) -> Community excitement	0.360	0.087	4.137	0.000
Nationalities (Arabs) -> Community excitement	0.226	0.088	2.574	0.010
Nationalities (Qatari) -> Community pride	0.145	0.100	1.442	0.149
Nationalities (Arabs) -> Community pride	0.155	0.099	1.563	0.118
Nationalities (Qatari) -> Enhanced community attachment	0.293	0.090	3.244	0.001
Nationalities (Arabs) -> Enhanced community attachment	0.123	0.088	1.398	0.162
Nationalities (Qatari) -> Event excitement	0.344	0.084	4.083	0.000
Nationalities (Arabs) -> Event excitement	0.172	0.081	2.139	0.032
Nationalities (Qatari) -> Pride to improve infrastructure	0.214	0.086	2.475	0.013
Nationalities (Arabs) -> Pride to improve infrastructure	0.104	0.087	1.195	0.232
**Subgroup by gender**				
Gender (Female) -> Community excitement	0.104	0.059	1.765	0.078
Gender (Female) -> Community pride	0.172	0.056	3.079	0.002
Gender (Female) -> Enhanced community attachment	0.122	0.062	1.968	0.049
Gender (Female) -> Event excitement	0.100	0.062	1.625	0.104
Gender (Female) -> Pride to improve infrastructure	0.163	0.059	2.748	0.006

B, Path coefficient; SE, Standard Error; t, Bootstrap t-value; p, p-value.

The reference group for the variable Nationalities is “Non-Arabs” and for variable gender is “Male”.

The purpose of this study was to measure the psychological and emotional impact (psychic income) perceived by subgroups of university students living in Qatar after hosting the 2019 IAAF Athletics World Championships. Students were classified into two different groups. The first group assorted students based on their nationalities. Therefore, three different categories based on their nationalities emerged, Qatari nationals for students with Qatari nationality, Arab nationals excluding the Qatari ones, and non-Arabs group. Then the second group categorized students based on their gender (see Figure 1).

Findings indicate that all five dimensions contribute significantly and positively to total psychological and emotional impact (psychic income). The highest significant effect was for enhanced community attachment (β_(enhanced community attachment) = 0.338, *p* < 0.001), followed by event excitement (β_(event excitement) = 0.259, *p* < 0.001), community pride (β_(community pride) = 0.221, *p* < 0.001), pride to improve infrastructure (β_(pride to improve infrastructure) = 0.210, *p* < 0.001), and community excitement (β_(community excitement) = 0.137, *p* < 0.001).

Furthermore, Table 2 shows a significant association between the nationalities and community excitement [β_(Nationalities (Qatari)] = 0.360, *p* < 0.001 and β_[Nationalities (Arabs) = 0.226, *p* = 0.010], indicating that on the community excitement dimension Qatari youth residents were more excited compared to Arab and non-Arab youth, and the same applies to Arab youth compared to non-Arab youth. On the other hand, there was no significance difference on the community pride between the nationalities of youth as *P*-values is higher than 0.05; [β_(Nationalities (Qatari)] = 0.145, *p* = 0.149 and β_[Nationalities (Arabs) = 0.155, *p* = 0.118].

Besides, these findings support the decision-makers/ organizers efforts in improving infrastructure and facilities around the city. Data shows that a significant difference between Qatari youth nationals and non-Arab youth nationals [β_(Nationalities (Qatari) = 0.214, *p* = 0.013], on the other hand there was no significant difference between Arab and non-Arab youth when it comes to assessing their pride to improve infrastructure, *P*-values higher then 0.05 {β_[Nationalities (Arabs)] = 0.104, *p* = 0.232}. Thus, it had a higher impact on Qatari youth compares to Arab and non-Arab youth.

In terms of enhanced community attachment, there were significant differences between Qatari and non-Arab youth {β_[Nationalities (Qatari)] = 0.293, *p* = 0.001}. On the other hand, the difference between Arab and non-Arab youth was not significant {β_[Nationalities (Arabs)] = 0.123, *p* = 0.162}, although Qatari youth felt more attached as a community compared to Arab youth, and Arab youth to non-Arab youth respectively. Perhaps community attachment is influenced by Qatari youth being nationals and hence feel more attached in comparison to Arab and non-Arab youth; whereas non-Arab youth feel less attached given that they do not share a common language and similar culture as opposed to Arab youth with Qatari youth.

Regarding the event excitement, there were significant differences between youth of different nationalities {β_[Nationalities (Qatari)] = 0.344, *p* < 0.001 and β_[Nationalities (Arabs)] = 0.172, p-value = 0.032}. This indicates that event excitement is experienced more by Qatari youth nationals compared to Arab youth nationals and non-Arab youth nationals. These results indicate that the perceived impact on nationality varies between the main five dimensions supporting H1.

This study extends the measurement to assess the perceived impact on gender. Results indicate that there were significant differences between female and male youth, thus supporting H2. Female youth compared to male youth felt prouder, more attached to the community, and prouder when it comes to infrastructure improvement. This can be reflected in the following results, “community pride” {β_[Gender (Female)] = 0.172, *p* = 0.002}, “enhanced community attachment” {β_[Gender (Female)] = 0.122, *p* = 0.049}, and “pride to improve infrastructure” {β_[Gender (Female)] = 0.163, *p* = 0.006}.

Next to that, there was there were no significant differences between male and female youth regarding excitement; “community excitement” {β_[Gender (Female)] = 0.104, *p* = 0.078}, and “event excitement” {β_[Gender (Female)] = 0.100, *p* = 0.104}.

## Discussion

Hosting sport events in the Arab World have attracted researchers to understand the motives behind hosting them. While many focused on the economic and political aspects, few have considered the impact on the community or targeted the social impact that can be generated. These events play an important role in bridging and bonding communities, enhancing pride, and increasing excitement (Crompton, 2004). Even if these aspects are felt, yet there is still a lack in quantifying them. This work gets one step closer by assessing the psychic income of university students residing in Qatar. Although hosting sport events might generate economic benefits to the host communities (Duglio and Beltramo, 2017)), this study confirms that intangible impact was significant, and indicates that the received impact on university students was positive. Following Kim and Walker (2012) approach the five factors were assessed, with the outcomes providing decision-makers and event organizers with a better measurement and understanding for added event impact amongst the youth in Qatar (see Table 2). Furthermore, this study extends to measure the impact associated with subgroups of the youth in Qatar in particular. This relative influence was consistent with prior work on the impact of hosting sport events considering different demographic variables (Truno, 1995; Kim and Petrick, 2005; Wicker and Sotiriadou, 2013; Al-Emadi et al., 2017, 2022).

The result of the current study also shows consistency with previous work, in relation to the community pride/image variable results, which indicates a positive outcome amongst respondents (Crompton, 2004; Karadakis and Kaplanidou, 2012; Kim and Walker, 2012; Hallmann et al., 2013; Gibson et al., 2014; Kim et al., 2015; Ishac et al., 2018). For example, Ghaderi et al. (2021) in their study assessing the psychic income on a small-scale sport event, found that the image of the host city is promoted among residents distinct from the size of the event. This work supports the government strategy in promoting the city's image. Hosting sport events have a great ability to promote the image of the host city and boost the sense of pride among youth residents, as highlighted in our study (see Table 2), and in previous work assessing the perceived impact of residents in general prior to (Al-Emadi et al., 2017, 2022), and post (Ishac et al., 2018) the hosting of international sport events in Qatar.

Furthermore, the local support generated within the community would provide decision-makers, organizers with the validations needed for bidding to host these major international sport events (Crompton, 2004). Hence, on the community pride Ghaderi et al. (2021) mentioned that pride cannot be measured in the short run; it might require more than a one-time event to strengthen it, and that might explain when assessing community pride in this study, there was not a significant difference between the three different youth groups.

Furthermore, the result shows that “attachment to the community,” was similar to previous work (Crompton, 2004; Kim and Walker, 2012; Kim et al., 2015; Liu, 2016; Ghaderi et al., 2021). This result shows that hosting sport events can boost community belonging and empowerment amongst the youth; therefore, it might be important for organizers to link these events to the local culture, this way it will help in preserving the traditional and the cultural aspect of the event (Ghaderi et al., 2021).

The last three factors, “event excitement,” “community infrastructure,” and “community excitement” show consistency with the findings of Kim and Walker (2012) and Liu (2016). In their previous study assessing the perceived impact among Qatari residents, Al-Emadi et al. (2017) and Al-Emadi et al. (2022), highlight that the socio-cultural factors are the most important elements that generated a positive impact within the Qatari residents. Their results show a positive attitude with residents being excited about hosting the FIFA World Cup. Similarly, the current study found that Qatari youth residents were more excited compared to Arab and non-Arab youth, and the same applies to Arab youth compared to non-Arab youth. Once again this result underscores that excitement about an event can be felt even if resident youth do not attend the event physically.

Moreover, the results indicate that there were significant differences between female and male youth in Qatar. The additional findings for gender differences amongst the youth are valuable for both theoretical advancements and practitioners. As a result, female youth participants associate a higher positive impact compared to male youth. This relative influence was consistent with prior work on the impact of hosting sport events considering different demographic variables (Truno, 1995; Kim and Petrick, 2005; Wicker and Sotiriadou, 2013). Following a study by Theodorakis et al. (2019), fans from the Middle East were highly involved with football, while males were more involved than females. Similarly, Al-Emadi et al. (2022) highlight that the context of football will generally attract more males compared to females given the more socio-cultural norms that govern Qatari society. While our study indicates a higher positive impact amongst the female youth in Qatar in relation to the 2019 IAAF World championships, in contrast Al-Emadi et al. (2022) highlighted that females in general have a higher concern regarding the hosting of the 2022 FIFA World Cup than males that could be due to some cultural challenges. Females tends to be more cultural conservative and had more concerns about the World Cup not respecting local culture in relation to football fan behavior, alcohol consumption and respecting local dress and public behavior (Al-Emadi et al., 2022). Interestingly, their study also showed that the younger age group were more concerned about alcohol consumption, which they attributed to them perhaps being more exposed to this behavior when attending sport events. Thus, while the two events may attract different audiences, both females and the youth have highlighted concerns that could be problematic during the World Cup if not managed appropriately.

Following a study comparing the psychic income prior and post the 2010 FIFA World Cup among residents of the host nation, the result significantly increased post-event (Gibson et al., 2014). Therefore, the results of this study can explain and project with a stronger or more positive impact to be generated from the 2022 FIFA World Cup especially since males generally are more involved in football. Since the majority of participants in this study also did not attend, yet a positive psychic income resulted also bodes well for generating positive results from the World Cup amongst female youth in particular. Moreover, this study indicates that event organizers should acquire or develop new methods that stimulate male youth, to improve their interest in different types of sport events beyond the football context, especially with Qatar establishing itself as a regional sport hub by attracting a diverse range of international sport events.

The relationship between nationalities and the other five dimensions assessed was a very interesting finding. Results show that within the community excitement dimension, the received impact on Arab youth is higher than non-Arab youth. This result can be triggered by factors not related to the nature of the sport events. Previous studies highlighted that the place where residents grew up influences their perceptions (Lankford, 1994; McCool and Martin, 1994; McGehee and Andereck, 2004). Therefore, the authors argue that Arab youth nationals born and raised in Qatar can perceive a higher positive impact than non-Arab youth nationals. Furthermore, they argue that exercising the blockade on Qatar by some of the neighboring countries (at the time this study was conducted) resulted in more psychological attachment by the Arab youth residents than the non-Arab youth residents.

This was reflected in the 2021 FIFA Arab Cup, the precursor to the 2022 FIFA World Cup, with 631,742 tickets sold where 89% were purchased by Qatari residents and 11% by international visitors (The Peninsula, 2022). Despite the instability within the region, adding to that the consequences of Covid-19 still Arabs living in Qatar and those outside came to support their national football teams (The Peninsula, 2021). Interestingly, Egypt, Tunisia and Jordan were among the top ticket buyers by nationality, while Egypt, Saudi Arabia and Iraq were part of the top ticket buyers by residents (The Peninsula, 2022). Travel bookings from neighboring countries such as Oman, Kuwait and Lebanon were also experienced (The Peninsula, 2021). Support by Arab nations for the World Cup is aligned to the positioning of the World Cup as representing the Arab World (Centre for International and Regional Studies, n.d.), thus this bodes well for achieving higher psychic income from the 2022 FIFA World Cup; for Arab nationals in general, and amongst the youth in particular.

## Conclusion

The study highlights that a positive impact was perceived by the Qatari youth residents who participated in this study when the 2019 IAAF Athletics World championships was hosted, despite the negative media reports about the lack of attendance by local residents. The findings seem to illustrate that even when resident youth do not attend events in person, hosting sport events can still be beneficial to society. This study covers an important part not highlighted previously in the literature. By evaluating the psychic income received within subgroups after the event took place, decision-makers will be able to modify their strategies to have a higher impact in reaching their objectives by assessing the cumulative impact of the hosting major international sport events. In our case, these results will provide a better projection for the implementation of the 2022 FIFA World Cup, and that will help in reaching Qatar 2030 vision objectives.

While these results can be replicated in similar communities, it is important to highlight that this study presents a unique sample since it focuses on measuring the impact on youth. Within its 2030 vision, Qatar is aiming for a long-term social impact; the country aims to provide with a high standard of living not only for today citizens but also for the future generation (Hukoomi, 2022b). Thus, measuring it requires studying the social impact on the young generation who will become the future of the country. The results remain limited in application to international sport events that have a lower number of fans and engaged residents compared to other sports events. Projecting to the 2022 FIFA World Cup, especially since the majority of fans from the region are interested in football and may unlikely be able to attend physically due to the limitations of purchasing tickets; this study highlights that regardless of attending the event physically or not, psychic income will be generated due to the interest that fans or residents show in football. Given that the World Cup is being positioned as one for the Arab region, taking local culture into consideration may further boost positive psychic income. The importance of repeating the study pre- and post the World Cup amongst the youth of Qatar is therefore underscored.

Another area of future research is to consider the changes happening on the macro level (Kim and Petrick, 2005); not only by evaluating the cultural and environmental aspects, additionally it is important to focus on other aspects. The past 5 years witnessed an unstable political situation within the Gulf region. The authors argue that this instability can affect or trigger the residents emotionally, and can skew the results. Furthermore, when it comes to Arabs and non-Arabs measuring the received impact from hosting sport events, it is important to take into consideration the stability in their home countries (e.g., economic and/or political). This will be reflected in their community engagement in the host city, as they refer to the host country as their home country and that will likely have an impact on their responses.

Future research is also needed to examine the psychic income generated within the Gulf region. Since Qatar will be hosting the 2022 FIFA World Cup, many residents within the region will engage in this event as the World Cup has been positioned to have a positive legacy not only for Qatar but for the MENA region, more broadly. Therefore, it will be important to measure whether hosting such an event has a greater positive impact that could be measured within residents of other countries within the Gulf or the whole MENA region. Assessing the psychic income that can be generated from hosting mega sport events can provide other Gulf countries with more insights since the majority of these countries are also investing heavily in hosting sport events. It can also help increase collaboration, and joining ventures to boost the impact generated and to have a higher impact within the Gulf society.

## Data availability statement

The raw data supporting the conclusions of this article will be made available by the authors, without undue reservation.

## Ethics statement

The studies involving human participants were reviewed and approved by Qatar University IRB Committee. All participants had to agree to consent in order to proceed with the study.

## Author contributions

All authors listed have made a substantial, direct, and intellectual contribution to the work and approved it for publication.

## Conflict of interest

The authors declare that the research was conducted in the absence of any commercial or financial relationships that could be construed as a potential conflict of interest.

## Publisher's note

All claims expressed in this article are solely those of the authors and do not necessarily represent those of their affiliated organizations, or those of the publisher, the editors and the reviewers. Any product that may be evaluated in this article, or claim that may be made by its manufacturer, is not guaranteed or endorsed by the publisher.
